# Supplementary material for: Decision to use denture adhesive in complete denture wearers after one-month run-in period: A quasi-experimental study
Source: PLoS One. 2022 Dec 1;17(12):e0276760. doi: 10.1371/journal.pone.0276760 (PMC9714889; doi:10.1371/journal.pone.0276760)
Supplement: S1 File — (PDF) [file pone.0276760.s003.pdf]

เอกสารข้อมูลคำอธิบายสำหรับอาสาสมัครที่เข้าร่วมในการวิจัย

(Patient/Participant Information Sheet)”

1. โครงการวิจัยเรื่อง ผลของกาวติดฟันเทียมที่มีต่อประสิทธิภาพการบดเคี้ยว แรงกัดฟันสูงสุด และคุณภาพชีวิตในมิติสุขภาพช่องปากในผู้ป่วยฟันเทียมทั้งปาก
2. ชื่อผู้วิจัย ทันตแพทย์หญิง บุษรา ทองย้อย

   อ.ทญ.ดร. ณฤดี ลิ้มปวงทิพย์

   ผศ.ทพ.ดร. วัชรศักดิ์ ตุมราศวิน

   สถาบันที่สังกัด ภาควิชาทันตกรรมประดิษฐ์ คณะทันตแพทยศาสตร์ จุฬาลงกรณ์มหาวิทยาลัย

   แหล่งทุนวิจัย คณะทันตแพทยศาสตร์ จุฬาลงกรณ์มหาวิทยาลัย

3. วัตถุประสงค์ของโครงการ
   - เพื่อศึกษาผลของกาวติดฟันเทียมต่อประสิทธิภาพการบดเคี้ยวในผู้ป่วยฟันเทียมทั้งปาก
   - เพื่อศึกษาผลของกาวติดฟันเทียมต่อแรงกัดฟันสูงสุดในผู้ป่วยฟันเทียมทั้งปาก
   - เพื่อศึกษาผลของกาวติดฟันเทียมต่อคุณภาพชีวิตในมิติสุขภาพช่องปากในผู้ป่วยฟันเทียมทั้งปาก
4. สถานที่ดำเนินการวิจัย

ภาควิชาทันตกรรมประดิษฐ์บัณฑิตศึกษา ชั้น 8 อาคารสมเด็จย่า 93 ปี คณะทันตแพทยศาสตร์ จุฬาลงกรณ์มหาวิทยาลัย

5. วิธีการที่เกี่ยวข้องกับการวิจัย

อาสาสมัครแต่ละคนจะได้รับการตรวจ 3 ครั้ง ดังนี้

ครั้งที่ 1

- ตรวจภายในช่องปาก สอบถามข้อมูลส่วนบุคคล เก็บข้อมูลจากแฟ้มประวัติ
- ตรวจคุณภาพฟันเทียมทั้งปากบนและล่าง
- ทดสอบประสิทธิภาพการบดเคี้ยว อาสาสมัครเคี้ยวถั่วลิสงปริมาณ 3 กรัม โดยกำหนดจำนวนรอบในการเคี้ยวถั่วลิสง คือ 10, 20, 30 รอบและ 40 วงเคี้ยว เมื่อเคี้ยวเสร็จในแต่ละรอบให้บ้วนถั่วลิสงใส่ภาชนะให้หมด กลั้วปากด้วยน้ำเปล่าแล้วบ้วนลงใส่ภาชนะอีกครั้ง ทันตแพทย์จะเป็นผู้ล้างถั่วที่ติดบนฟันปลอมและเหงือกในการเคี้ยวแต่ละครั้งออกให้ หลังจากเคี้ยวแต่ละรอบมีเวลาพัก 10 นาที
- ทดสอบแรงกัดฟันสูงสุด จำนวน 3 ครั้ง โดยกัดค้างไว้ 5 วินาที หลังจากกัดแต่ละครั้งมีเวลาพัก 5 นาที

- ให้สัมภาษณ์เกี่ยวกับผลกระทบที่เกิดขึ้นจากฟันเทียมต่อการใช้ชีวิจประจำวัน

จากนั้นอาสาสมัครแต่ละคนจะได้รับกาวติดฟันเทียมชนิดครีม (ยี่ห้อ Polident) ใช้ติดฟันเทียมทั้งปากบนและล่าง 1 ครั้งในตอนเช้าและใส่ฟันเทียมวันละ 8 ชั่วโมงในเวลากลางวัน ทุกวัน โดยเปลี่ยนและล้างกาวติดฟันเทียมทุกวัน เป็นเวลา 1 เดือน ทั้งบริเวณใต้ฐานฟันปลอมและเหงือก

ครั้งที่ 2 (หลังจากใช้กาวติดฟันเทียมมาแล้ว 1 เดือน)

ในทุกขั้นตอนของการทดสอบครั้งนี้ ทำภายใต้การใช้กาวติดฟันปลอม

- ทดสอบประสิทธิภาพการบดเคี้ยว โดยให้อาสาสมัครเคี้ยวถั่วลิสงปริมาณ 3 กรัม โดยกำหนดจำนวนรอบในการเคี้ยวถั่วลิสง 10, 20, 30 และ 40 วงเคี้ยว เมื่อเคี้ยวเสร็จในแต่ละรอบให้บ้วนถั่วลิสงใส่ภาชนะให้หมด กลั้วปากด้วยน้ำเปล่าแล้วบ้วนลงใส่ภาชนะอีกครั้ง หลังจากเคี้ยวแต่ละรอบมีเวลาพัก 10 นาที เพื่อทำความสะอาดถั่ว และกาวติดฟันปลอมที่ติดใต้ฐานฟันปลอม และเหงือก
- ทดสอบแรงกัดฟันสูงสุด จำนวน 3 ครั้ง โดยกัดค้างไว้ 5 วินาที หลังจากกัดแต่ละครั้งมีเวลาพัก 5 นาที
- ให้สัมภาษณ์เกี่ยวกับผลกระทบที่เกิดขึ้นจากฟันเทียมต่อการใช้ชีวิตประจำวัน

ในครั้งนี้ อาสาสมัครจะเป็นผู้เลือกว่าต้องการใช้ หรือไม่ใช้กาวติดฟันเทียมต่อ แล้วใช้ / ไม่ใช้กาวติดฟันเทียมต่ออีก 1 เดือน

ครั้งที่ 3 (หลังจากอาสาสมัครตัดสินใจใช้ หรือไม่ใช้กาวติดฟันเทียมมาแล้ว 1 เดือน)

กรณีที่อาสาสมัครเลือกใช้กาวติดฟันเทียมต่อ จะทดสอบภายใต้การใช้กาวติดฟันเทียม แต่ในกรณีที่อาสาสมัครเลือกไม่ใช้กาวติดฟันเทียมต่อ จะทดสอบโดยไม่ใช้กาวติดฟันเทียม

- ทดสอบประสิทธิภาพการบดเคี้ยว โดยให้อาสาสมัครเคี้ยวถั่วลิสงปริมาณ 3 กรัม โดยกำหนดจำนวนรอบในการเคี้ยวถั่วลิสง 10, 20, 30 และ 40 วงเคี้ยว เมื่อเคี้ยวเสร็จในแต่ละรอบให้บ้วนถั่วลิสงใส่ภาชนะให้หมด กลั้วปากด้วยน้ำเปล่าแล้วบ้วนลงใส่ภาชนะอีกครั้ง หลังจากเคี้ยวแต่ละรอบมีเวลาพัก 10 นาที
- ทดสอบแรงกัดฟันสูงสุด จำนวน 3 ครั้ง หลังจากกัดแต่ละครั้งมีเวลาพัก 5 นาที
- ให้สัมภาษณ์เกี่ยวกับผลกระทบที่เกิดขึ้นจากฟันเทียมต่อการใช้ชีวิตประจำวัน

หลังจากสิ้นสุดโครงการ ผู้ที่มีฟันเทียมคุณภาพไม่เหมาะสม จะได้รับการทำฟันเทียมชุดใหม่จากนิสิตคณะทันตแพทยศาสตร์ จุฬาลงกรณ์มหาวิทยาลัย ภายในการดูแลของคณาจารย์ภาควิชาทันตกรรมประดิษฐ์

6. เหตุผลที่เชิญเข้าร่วมเป็นอาสาสมัครในโครงการ

เนื่องจากท่าน

- ใส่ฟันเทียมทั้งปากบนและล่างมาแล้วอย่างน้อย 6 เดือน
- ฟันเทียมหลวมแต่ไม่เคยใช้กาวติดฟันเทียม
- ไม่ได้รับการปักรากเทียมใต้ฟันเทียมทั้งปากบนและล่าง
- ไม่มีประวัติแพ้ถั่วลิสงและประวัติแพ้สารประกอบของกาวติดฟันเทียม
- ไม่มีข้อจำกัดทางด้านการเคลื่อนไหวของร่างกาย

7. ความรับผิดชอบของอาสาสมัคร และ ระยะเวลาที่อาสาสมัครจะอยู่ในโครงการ

ขอให้ท่านปฏิบัติตามที่ผู้วิจัยแนะนำ โดยใช้กาวติดฟันเทียมทั้งปากบนและล่าง 1 ครั้งในตอนเช้าและใส่ฟันเทียมวันละ 8 ชั่วโมงในเวลากลางวันทุกวัน โดยเปลี่ยนและล้างกาวติดฟันเทียมทุกวัน ด้วยขนแปรงนุ่มและน้ำสบู่ ทำความสะอาดทั้งในช่องปากและฟันเทียม โดยระยะเวลาที่อาสาสมัครจะอยู่ในโครงการ 2 เดือน

8. ประโยชน์ของการวิจัยที่อาสาสมัครและ/หรือผู้อื่นที่อาจได้รับ

ได้ทดลองใช้กาวติดฟันเทียมโดยไม่เสียค่าใช้จ่าย

ค่าเดินทาง 3 ครั้ง ครั้งละ 200 บาท

9. ความเสี่ยงหรือความไม่สะดวกที่อาจจะเกิดขึ้นแก่อาสาสมัคร และในบางกรณีแก่ทารกในครรภ์ หรือทารกที่ดื่มนมมารดา

- ในการเข้าร่วมโครงการวิจัยนี้อาสาสมัครต้องเดินทางมาเข้าร่วมเก็บข้อมูล 3 ครั้ง ในระยะเวลาที่กำหนด (2 เดือน) โดยในแต่ละครั้งใช้เวลาประมาณ 2 ชั่วโมง

- การทดสอบประสิทธิภาพการบดเคี้ยวโดยการเคี้ยวถั่วลิสงตามจำนวนรอบที่กำหนด อาจทำให้เกิดการเมื่อยล้าของกล้ามเนื้อบดเคี้ยวบริเวณใบหน้าได้

10. ค่าใช้จ่ายที่อาสาสมัครจะต้องจ่าย หรืออาจจะต้องจ่าย

อาสาสมัครไม่ต้องออกค่าใช้จ่ายใดๆ

11. การชดเชยใดๆ และการรักษาที่จะจัดให้แก่อาสาสมัครในกรณีที่ได้รับอันตรายซึ่งเกี่ยวข้องกับการวิจัย

หากท่านได้รับอันตรายจากการทำวิจัย ผู้วิจัยจะดำเนินการให้ท่านได้รับการรักษาโดยผู้วิจัยจะเป็นผู้รับผิดชอบค่าใช้จ่ายของการรักษา

12. การจ่ายค่าเดินทาง ค่าเสียเวลา แก่อาสาสมัครที่เข้าร่วมในการ

อาสาสมัครจะได้รับค่าเดินทาง 3 ครั้ง ครั้งละ 200 บาท และอาสาสมัครจะได้รับกาวติดฟันเทียมในครั้งแรกทุกคน แต่ครั้งที่สองของการเข้าร่วม หากอาสาสมัครเลือกใช้กาวติดฟันปลอมต่อ จะได้รับกาวติดฟันเทียมอีกในครั้งที่สอง

13. เหตุการณ์ที่อาจจะเกิดขึ้น หรือเหตุผลซึ่งผู้วิจัยจะต้องยกเลิกการเข้าร่วมในโครงการวิจัยของอาสาสมัคร

กรณีที่อาสาสมัครไม่สามารถเข้าร่วมเก็บข้อมูลทั้ง 3 ครั้ง ในระยะเวลาที่กำหนด (2 เดือน) ผู้วิจัยจะขอยกเลิกการเข้าร่วมโครงการวิจัยของอาสาสมัคร

14. มีการเก็บชิ้นตัวอย่างที่ได้มาจากอาสาสมัครเอาไว้ใช้ในโครงการวิจัยในอนาคตหรือไม่ เก็บจำนวนเท่าไหร่ อย่างไร และที่ไหน

ไม่มี
