# Supplementary material for: Decision to use denture adhesive in complete denture wearers after one-month run-in period: A quasi-experimental study
Source: PLoS One. 2022 Dec 1;17(12):e0276760. doi: 10.1371/journal.pone.0276760 (PMC9714889; doi:10.1371/journal.pone.0276760)
Supplement: S2 File — (PDF) [file pone.0276760.s004.pdf]

Patient/Participant Information Sheet

1. Project title: Effect of denture adhesive on masticatory performance, maximum bite force, and oral health-related quality of life of complete denture wearers
2. Investigators Dr.Budsara Thongyoi

   Dr.Nareudee Limpuangthip

   Assoc Prof.Wacharasak Tumrasvin

   Institution Department of Prosthodontics, Faculty of Dentistry, Chulalongkorn University

   Funding source(s) Research fund, Faculty of Dentistry, Chulalongkorn University

3. Project objectives

- To study the effect of denture adhesive on masticatory performance of complete denture wearers
- To study the effect of denture adhesive on maximum bite force of complete denture wearers
- To study the effect of denture adhesive on oral health-related quality of life of complete denture wearers

4. Place of data collection

   Postgraduate Prosthodontic Clinic, 8th floor, Somdeja 93-year building, Faculty of Dentistry, Chulalongkorn University

5. Methodology

   Each participant will undergo the following examination.

   1st visit:

   - Oral examination, Interview for personnel information, chart record review
   - Denture quality evaluation
   - Masticatory performance evaluation: Participant chews 3 grams of peanut with the indicated numbers of chewing stroke; 10, 20, 30 and 40 chewing strokes. After chewing, the participant splits the comminuted peanut particles in the given container, gargling the

mouth with tap water, and split the remaining comminuted peanut particles in the given container. The peanut particles attached on the denture and gum will be removed by the dentist. The resting interval for each chewing test is 10 minutes.

- Bite force evaluation: Participant bites with a maximum force on the given sheet placed in the mouth and holds in the maximal intercuspal position for 5 second. The resting interval for each chewing test is 5 minutes.
- Participant will be interviewed about the oral impact caused by denture on the ability to perform daily activities.

Then, the participant will receive denture adhesive (Polident) for applying on both maxillary and mandibular denture once a day in the morning. After the denture has worn for 8 hours a day, the denture adhesive must be removed in the evening. The denture adhesive must be removed from the denture and gum. This procedure is performed daily for 1 month period.

2nd Visit (After 1 month of denture adhesive use)

The following test will be performed when using denture adhesive

- Masticatory performance evaluation: Participant chews 3 grams of peanut with the indicated numbers of chewing stroke; 10, 20, 30 and 40 chewing strokes. After chewing, the participant splits the comminuted peanut particles in the given container, gargling the mouth with tap water, and split the remaining comminuted peanut particles in the given container. The peanut particles attached on the denture and gum will be removed by the dentist. The resting interval for each chewing test is 10 minutes.
- Bite force evaluation: Participant bites with a maximum force on the given sheet placed in the mouth and holds in the maximal intercuspal position for 5 second. The resting interval for each chewing test is 5 minutes.
- Participant will be interviewed about the oral impact caused by denture on the ability to perform daily activities.

At this visit, the participant has to choose whether he/she wants to continue using denture adhesive for 1-month period.

3rd Visit (One -month after the participant continues/discontinues using denture adhesive)

These following procedures will be performed with or without denture adhesive, depended on the participants decision for the continuation of denture adhesive use.

- Masticatory performance evaluation: Participant chews 3 grams of peanut with the indicated numbers of chewing stroke; 10, 20, 30 and 40 chewing strokes. After chewing, the participant splits the comminuted peanut particles in the given container, gargling the mouth with tap water, and split the remaining comminuted peanut particles in the given container. The peanut particles attached on the denture and gum will be removed by the dentist. The resting interval for each chewing test is 10 minutes.
- Bite force evaluation: Participant bites with a maximum force on the given sheet placed in the mouth and holds in the maximal intercuspal position for 5 second. The resting interval for each chewing test is 5 minutes.
- Participant will be interviewed about the oral impact caused by denture on the ability to perform daily activities.

At the end of the project, the participants with unacceptable denture quality will undergo a new denture fabrication by dental students of the Faculty of Dentistry, Chulalongkorn University under a supervision of the staff of prosthodontic department.

6. Reasons for the invitation to participate the project

Since the participants have the following inclusion criteria:

- Has been wearing the maxillary and mandibular denture for at least 6 months
- Has ill-fitting denture but never tried denture adhesive use
- Has never done an implant-assist overdenture
- No history of allergic to peanut and any components in denture adhesive
- Absence of physical limitation

7. Duty of the participant and retention period in the project

The participant has to follow the dentist’s recommendation as follows: apply denture adhesive on the upper and lower denture once a day in the morning. After use the denture for up to 8 hours, the denture adhesive must be removed from the mouth and denture using soft toothbrush and liquid soap. A total duration of the project is 2 months.

8. Benefits to the participants and/or others

- Trying denture adhesive use without any payment
- The participant will receive 200 baht for each visit of participation as travelling expense

9. Possible risk(s) or inconvenience to the participant, in some cases, to the fetus or infant who receives breastfeeding

- The participant has to travel and visit the clinic 3 times for data collection during 2-month period. It takes about 2 hours per visit.
- The participant has to perform the mastication and bite force test which may cause facial muscle fatigue.

10. Additional expenses / Possible additional expenses

No

11. Any compensation and treatment in case of any accidents to the participant caused by the intervention during the project

If any accident occurs or treatment is required, the investigator will be responsible for all expenses.

12. Expenses for travelling and time cost for project participation

The participant will receive 200 baht for each visit of participation as travelling expense. Denture adhesive will be provided without any out-of-pocket payment. If the participant is willing for continue using denture adhesive, he/she will receive one more tube of denture adhesive.

13. Any possible incident or reasons for the participant’s withdrawal

If the participant cannot attend the clinic for data collection within 2-month period, he/she will be withdrawn from the study.

14. Any specimen collection for further study. If yes, please indicate the amount, place for specimen collection and storage methods.

    No
