# Supplementary material for: Decision to use denture adhesive in complete denture wearers after one-month run-in period: A quasi-experimental study
Source: PLoS One. 2022 Dec 1;17(12):e0276760. doi: 10.1371/journal.pone.0276760 (PMC9714889; doi:10.1371/journal.pone.0276760)
Supplement: Attachment — Submitted filename: PONE-D-22-08584_reviewer.pdf [file pone.0276760.s005.pdf]

PLOS ONE

Decision to Use Denture Adhesive in Complete Denture Wearers After One-Month Trial: A Quasi-Experimental Study

--Manuscript Draft--

**Manuscript Number:**	PONE-D-22-08584
**Article Type:**	Clinical Trial
**Full Title:**	Decision to Use Denture Adhesive in Complete Denture Wearers After One-Month Trial: A Quasi-Experimental Study
**Short Title:**	Decision to use denture adhesive
**Corresponding Author:**	Wacharasak Tumrasvin, DDS, PhD<br>Chulalongkorn University Faculty Of Dentistry<br>Pathumwan, Bangkok THAILAND
**Keywords:**	complete denture; denture adhesive, oral health related quality of life; masticatory function; masticatory performance; ill fit denture
**Abstract:**	Objectives<br><br>This study aimed to assess two patient-based outcomes of CD wearers who continued or discontinued using DA after one-month trial use of DA.<br><br>Methods<br><br>This quasi-experimental study included 76 CD wearers. Two patient-based outcomes were oral health-related quality of life and masticatory performance, determined by the Thai-version of oral impacts on daily performances index and multiple sieve method of 20-stroke peanut mastication. Denture retention and stability were evaluated using the CU-modified Kapur criteria to classify CD into acceptable or unacceptable quality. The outcomes were collected at 3 time points: 1) at baseline (T0), 2) after 1-month trial of DA use (T1), at which time the participants decided whether to continue using DA, and 3) 1-month after continued or discontinued using DA (T2). Changes in the percentages of having an oral impact were evaluated using the McNemar and chi-squared or Fisher's Exact tests, while the changes in peanut particle size were evaluated using the independent t-test and repeated ANOVA. Multiple logistic regression was used to analyze the factors associated with deciding to continue using DA.<br><br>Results<br><br>Participants who continued using DA at T2 had greater oral impact reduction after 1-month trial of DA use, whereas those who discontinued using had emerged cleaning and emotional impacts. At T1 and T2, peanut particle size of the participants who continued and discontinued using DA was not significantly changed from T0, except the acceptable CD wearers who discontinued using DA at T2 whose peanut particle size was decreased from T0 to T2.<br><br>Conclusions<br><br>Baseline oral impacts and its change influenced CD wearers' decision whether to continue using DA, while masticatory performance showed no influence on patients' decision.
**Order of Authors:**	Nareudee Limpuangthip
	Wacharasak Tumrasvin, DDS, PhD
	Budsara Thongyoi
**Additional Information:**	
**Question**	**Response**
**Financial Disclosure**	The present research was funded by the Faculty Research Grant, Faculty of Dentistry,

Powered by Editorial Manager® and ProduXion Manager® from Aries Systems Corporation

Enter a financial disclosure statement that describes the sources of funding for the work included in this submission. Review the submission guidelines for detailed requirements. View published research articles from *PLOS ONE* for specific examples.<br><br>This statement is required for submission and **will appear in the published article** if the submission is accepted. Please make sure it is accurate.<br><br>**Unfunded studies**<br>Enter: *The author(s) received no specific funding for this work.*<br><br>**Funded studies**<br>Enter a statement with the following details:<br>• Initials of the authors who received each award<br>• Grant numbers awarded to each author<br>• The full name of each funder<br>• URL of each funder website<br>• Did the sponsors or funders play any role in the study design, data collection and analysis, decision to publish, or preparation of the manuscript?<br>• **NO** - Include this sentence at the end of your statement: *The funders had no role in study design, data collection and analysis, decision to publish, or preparation of the manuscript.*<br>• **YES** - Specify the role(s) played.<br><br>* typeset	Chulalongkorn University [grant number DRF 63002]. The funders had no role in study design, data collection and analysis, decision to publish, or preparation of the manuscript.
**Competing Interests**<br><br>Use the instructions below to enter a competing interest statement for this submission. On behalf of all authors, disclose any competing interests that could be perceived to bias this work—acknowledging all financial support and any other relevant financial or non-financial competing interests.<br><br>This statement is required for submission and **will appear in the published article** if	The authors declare that there is no potential conflict of interest in this study.

Powered by Editorial Manager® and ProduXion Manager® from Aries Systems Corporation

the submission is accepted. Please make sure it is accurate and that any funding sources listed in your Funding Information later in the submission form are also declared in your Financial Disclosure statement.<br><br>View published research articles from *PLOS ONE* for specific examples.<br><br>**NO authors have competing interests**<br><br>Enter: *The authors have declared that no competing interests exist.*<br><br>**Authors with competing interests**<br><br>Enter competing interest details beginning with this statement:<br><br>*I have read the journal's policy and the authors of this manuscript have the following competing interests: [insert competing interests here]*<br><br>* typeset	
**Ethics Statement**<br><br>Enter an ethics statement for this submission. This statement is required if the study involved:<br><br>• Human participants<br>• Human specimens or tissue<br>• Vertebrate animals or cephalopods<br>• Vertebrate embryos or tissues<br>• Field research<br><br>Write "N/A" if the submission does not require an ethics statement.<br><br>General guidance is provided below. Consult the submission guidelines for detailed instructions. **Make sure that all information entered here is included in the Methods section of the manuscript.**	The study was registered with Clnicaltrials.gov (Trial registration ID: NCT04942262). The study protocol was approved by the Human Research Ethics Committee of the Faculty of Dentistry, Chulalongkorn University (HREC-DCU 2019-068), and was conducted in accordance with the Declaration of Helsinki. All participants agreed to and signed the written informed consent prior to study participation.

Powered by Editorial Manager® and ProduXion Manager® from Aries Systems Corporation

**Format for specific study types**<br><br>**Human Subject Research (involving human participants and/or tissue)**<br>• Give the name of the institutional review board or ethics committee that approved the study<br>• Include the approval number and/or a statement indicating approval of this research<br>• Indicate the form of consent obtained (written/oral) or the reason that consent was not obtained (e.g. the data were analyzed anonymously)<br><br>**Animal Research (involving vertebrate animals, embryos or tissues)**<br>• Provide the name of the Institutional Animal Care and Use Committee (IACUC) or other relevant ethics board that reviewed the study protocol, and indicate whether they approved this research or granted a formal waiver of ethical approval<br>• Include an approval number if one was obtained<br>• If the study involved *non-human primates*, add *additional details* about animal welfare and steps taken to ameliorate suffering<br>• If anesthesia, euthanasia, or any kind of animal sacrifice is part of the study, include briefly which substances and/or methods were applied<br><br>**Field Research**<br><br>Include the following details if this study involves the collection of plant, animal, or other materials from a natural setting:<br>• Field permit number<br>• Name of the institution or relevant body that granted permission	
**Data Availability**<br><br>Authors are required to make all data underlying the findings described fully available, without restriction, and from the time of publication. PLOS allows rare exceptions to address legal and ethical concerns. See the PLOS Data Policy and FAQ for detailed information.	Yes - all data are fully available without restriction

Powered by Editorial Manager® and ProduXion Manager® from Aries Systems Corporation

A Data Availability Statement describing where the data can be found is required at submission. Your answers to this question constitute the Data Availability Statement and **will be published in the article**, if accepted.<br><br>**Important:** Stating 'data available on request from the author' is not sufficient. If your data are only available upon request, select 'No' for the first question and explain your exceptional situation in the text box.<br><br>Do the authors confirm that all data underlying the findings described in their manuscript are fully available without restriction?	
**Describe where the data may be found in full sentences. If you are copying our sample text, replace any instances of XXX with the appropriate details.**<br><br>• If the data are **held or will be held in a public repository**, include URLs, accession numbers or DOIs. If this information will only be available after acceptance, indicate this by ticking the box below. For example: *All XXX files are available from the XXX database (accession number(s) XXX, XXX.).*<br>• If the data are all contained **within the manuscript and/or Supporting Information files**, enter the following: *All relevant data are within the manuscript and its Supporting Information files.*<br>• If neither of these applies but you are able to provide **details of access elsewhere**, with or without limitations, please do so. For example:<br><br>*Data cannot be shared publicly because of [XXX]. Data are available from the XXX Institutional Data Access / Ethics Committee (contact via XXX) for researchers who meet the criteria for access to confidential data.*<br><br>*The data underlying the results presented in the study are available from (include the name of the third party*	All relevant data are within the paper and the supporting information file.

Powered by Editorial Manager® and ProduXion Manager® from Aries Systems Corporation

and contact information or URL).<br>• This text is appropriate if the data are owned by a third party and authors do not have permission to share the data.<br><br>* typeset	
Additional data availability information:	Tick here if your circumstances are not covered by the questions above and you need the journal's help to make your data available.

Title page

Decision to Use Denture Adhesive in Complete Denture Wearers After One-Month Trial: A Quasi-Experimental Study

Short title: Decision to use denture adhesive

Nareudee Limpuangthip[1¶], Wacharasak Tumrasvin[1¶*], Budsara Thongyoi[2]

[1]Department of Prosthodontics, Faculty of Dentistry, Chulalongkorn University, Bangkok, Thailand.

[2]Sikhoraphum Hospital, Surin 32110, Thailand.

[¶]These authors equally contributed to this study

*** Corresponding author:**

E-mail: wacharasak.t@chula.ac.th

Abstract

Objectives: This study aimed to assess two patient-based outcomes of CD wearers who continued or discontinued using DA after one-month trial use of DA.

Methods: This quasi-experimental study included 76 CD wearers. Two patient-based outcomes were oral health-related quality of life and masticatory performance, determined by the Thai-version of oral impacts on daily performances index and multiple sieve method of 20-stroke peanut mastication. Denture retention and stability were evaluated using the CU-modified Kapur criteria to classify CD into acceptable or unacceptable quality. The outcomes were collected at 3 time points: 1) at baseline (T0), 2) after 1-month trial of DA use (T1), at which time the participants decided whether to continue using DA, and 3) 1-month after continued or discontinued using DA (T2). Changes in the percentages of having an oral impact were evaluated using the McNemar and chi-squared or Fisher's Exact tests, while the changes in peanut particle size were evaluated using the independent t-test and repeated ANOVA. Multiple logistic regression was used to analyze the factors associated with deciding to continue using DA.

Results: Participants who continued using DA at T2 had greater oral impact reduction after 1-month trial of DA use, whereas those who discontinued using had emerged cleaning and emotional impacts. At T1 and T2, peanut particle size of the participants who continued and discontinued using DA was not significantly changed from T0, except the acceptable CD wearers who discontinued using DA at T2 whose peanut particle size was decreased from T0 to T2.

Conclusions: Baseline oral impacts and its change influenced CD wearers’ decision whether to continue using DA, while masticatory performance showed no influence on patients’ decision.

Introduction

People with edentulism are prevalent across countries, predominantly older people. Many of them need complete denture (CD) treatment to improve their masticatory ability and quality of life [1]. A proportion of CD wearers report problems after denture use, mostly due to poor denture retention and stability [2-4]. To solve these problems, some CD wearers use a denture adhesive (DA) to improve the denture fit, comfort, confidence, and masticatory ability [2, 5]. However, other CD wearers do not need to use DA because they can manage their denture well, and DA use does not significantly improve the denture fit or their masticatory ability [2, 5].

The efficiency of DA use has been identified using patient-based outcomes. They can be determined subjectively through patients' perception, and objectively through professional evaluation and quantitative measures of masticatory ability. A recent systematic review revealed that DA improves denture retention and masticatory performance of CD wearers [6]. Several studies reported that, after DA use, CD wearers were more satisfied with their denture function [7-9], or reported better oral health-related quality of life (OHRQoL) [10], which reflects patients' ability to perform physical, psychological, and social activities. However, conflicting results have been reported. Some authors reported that DA use did not always enhance masticatory performance [11], general satisfaction, or the overall OHRQoL of CD wearers [12]. Mostly, the investigators provided DA to the patients without providing a trial period of DA use to allow them making decision whether to continue using DA [7-9, 11, 12].

According to the surveys of DA use in edentulous populations, CD wearers use DA more frequently compared with removable partial denture wearers [13]. Some denture wearers have

never tried using DA, some had tried, but discontinued its use, and the others currently use a DA [2, 14]. The denture wearers who had never tried using DA was because they did not know that DAs are available. The first experience of DA use by most denture wearers was due to their dentist's recommendation, and patient's knowledge of DA that depended on information given by a dentist [2, 14]. Dentists recommend DA use, to both new and existing denture wearers, based on their experience and beliefs that DA provides physical and psychosocial benefits for their patients [15, 16].

Previous cross-sectional surveys have identified the factors associated with DA use in CD wearers [2, 13, 17]. Since DA experience could be an important factor for CD wearers to decide whether to use DA, the present study provided a 1-month trial of DA use for all CD wearers prior to choosing whether to continue using DA. The objective of this study was to assess the OHRQoL and masticatory performance of CD wearers who continued and discontinued using DA after a 1-month trial of DA use.

Materials and Methods

This present study design was a quasi-experimental study. The study was registered with Clnicaltrials.gov (Trial registration ID: NCT04942262). The study protocol was approved by the Human Research Ethics Committee of the Faculty of Dentistry, Chulalongkorn University (HREC-DCU 2019-068), and was conducted in accordance with the Declaration of Helsinki. All participants agreed to and signed the written informed consent prior to study participation. The study was written following the CONSORT guideline.

Participants

The eligible participants were CD wearers randomly recruited from patients who visited the Prosthodontic clinic at the Faculty of Dentistry, Chulalongkorn University for maintenance recall during January to December 2018. The inclusion criteria were patients who had worn removable maxillary and mandibular CDs for at least 1 year and were willing to improve their denture fit by trying DA use. The patients who wore a metal-based denture, had a history of DA use, had difficulty in responding to the interview, or were unwilling to follow the study protocol were excluded from the study.

A pilot study was performed to assess the reproducibility of the study protocol and to determine a sample size of the study. The sample size was estimated using the GPower v.3.1 program based on the hypothesis of two independent proportions. It was found that the percentages of participants with overall oral impact reduction after 1-month of DA use between those who decided to continue and discontinue using DA were 80% (n_1 = 14) and 48% (n_2 = 11), respectively. Including 10% drop-out rate, approximately 38 ~~samples~~ per group was required to achieve an 80% power at 5% significance level. Therefore, a total of 76 participants enrolled the study.

At baseline (T0), the background characteristics of the participants were collected, including general sociodemographic characteristics, CD age, and CD experiences. Oral and radiographic examinations were performed to determine the severity of the edentulous condition according to the American College of Prosthodontics (ACP) classification [18]. CD retention and stability were assessed by one of our investigators who was an experienced prosthodontist (W.T.) following the CU-modified Kapur criteria [3]. Then, the participants were classified in to

the CD wearers with acceptable or unacceptable denture quality. The intra-examiner reliability of CD retention and stability was determined by re-evaluating 15 CD wearers after 1 month, giving a Kappa score of 0.95.

Outcomes

One of our investigators was responsible for outcome determination (B.T). The primary outcome was OHRQoL determined using the Thai-version of Oral Impacts on Daily Performances (OIDP) index [19, 20]. The participants were interviewed about whether they had difficulties in performing the following 8 daily activities: eating, speaking and pronouncing, cleaning denture and mouth, sleeping, smiling, emotional stability, social contact, and carrying out work or housework. The frequency and severity of each impact were rated using a five-point Likert scale, and their multiplication gave a condition-specific (CS) impact score. The overall oral impact score was the sum of all 8 activity scores. The prevalence of an oral impact was categorized into absence (score = 0) or presence of an oral impact (score > 0).

The secondary outcome was masticatory performance assessed using the multiple sieve method of peanut mastication [3, 21]. The participants masticated 3 grams of roasted peanuts for 20 strokes in triplicate with a 10-minute resting interval between each test. The comminuted peanut particles were dried and sieved through 12 standard test sieves (Test sieve; Retcsh Technology GmbH) on a vibrating sieve shaker. A simple linear regression was plotted between the cumulative weight and diameter of each sieve test. The median peanut particle size (mm) was the sieve diameter through which 50% of the comminuted peanut particles passed. A smaller peanut particle size reflected a higher masticatory performance.

Intervention

At baseline (T0), all participants underwent a daily 1-month trial of DA use. They were provided a cream-type DA (Polident®, GlaxoSmithKline, Ireland) to all participants, and instructed to apply DA onto the tissue surface of their maxillary and mandibular dentures using a spot method [22]. The participants had to remove the DA and clean the denture every day after the last meal by soaking and brushing the CD with liquid soap and a soft toothbrush under running tap water [23]. Two gauze pads were used to remove DA from the denture and oral mucosa. The participants were provided a daily calendar for making a mark after DA application on each day. One external dentist also monitored participants' compliance by weekly telephone notification.

The OHRQoL, masticatory performance, and CD quality were evaluated at 3 time points by one of the investigators (B.T.):

1) T0, at baseline before using the DA. The baseline information regarding the outcomes were evaluated. Then, the participants then underwent a daily 1-month trial of DA use,

2) T1 (T0 + 1 month), after the 1-month trial period of DA use. The outcomes were evaluated with all participants using DA. The external prosthodontist asked the participants to choose whether they wanted to continue or discontinue using DA, and

3) T2 (T0 + 2 months), at 1-month after continuing or discontinuing DA use. At this time, some participants used DA during the outcome evaluations, while some did not, depending on the patient's decision on DA use.

Any complications caused by DA use were recorded. After the study ended, DA use in the participants with unacceptable denture quality were prohibited, and all of them underwent a new denture fabrication by dental students at the faculty.

Statistical analysis

Data were analyzed using STATA version 13.0 (STATA, Chicago, IL) at 5% significance level. The baseline characteristics of the participants who continued and discontinued using DA at T2 were described, and their associations were assessed using the chi-squared or independent t-test. The changes in the percentages of having an oral impact between time points and between the two groups were evaluated using the McNemar and chi-squared or Fisher's Exact tests, respectively. The changes in peanut particle size between the two groups and between time points were evaluated using the independent t-test and repeated one-way analysis of variance (ANOVA), respectively. Adjusting for patients' characteristics and CD experience, multiple logistic regression was used to analyze the factors associated with deciding to continue using DA. The associations between peanut particle size and eating impact score at each time point were assessed using Spearman's rank correlation.

Results

The flow diagram of the present study is illustrated in Figure 1. This study had 100% retention rate. Therefore, the data analysis comprised 76 participants. Approximately 47% of the participants decided to continue using DA after the 1-month trial using DA. The decision to

continue using DA was not significantly associated with sociodemographic characteristics, CD age, CD experience, or ACP classification; however, it was significantly associated with the presence or absence of a baseline oral impact (Table 1). Complications reported by two participants who discontinued using DA were due to feeling nausea and vomiting.

Figure 1. Flow diagram of the study (adopted from CONSORT diagram).

Table 1. Baseline characteristics of the participants at T0 (N = 76).

Variables	Total (%)	Decision to use DA at T2 (%)		p-value†
		Continue using (n=36)	Discontinue using (n=40)	
Age (years): mean ±SD	68.0 ±8.0	69.4 ±7.7	68.2 ±7.2	.51
Sex: male	56.6	66.8	47.5	.10
female	43.4	33.3	52.5	
Education: none to primary	60.5	58.7	67.5	.28
up to secondary	30.3	47.8	27.5	
up to tertiary	9.2	28.6	5.0	
Working status: employed	26.3	25.0	27.5	.80
unemployed	73.7	75.0	72.5	
Having CD experience: yes	46.0	44.4	47.5	.79
no	54.0	55.6	52.5	
CD age (years): mean ±SD	2.6 ±1.8	2.3 ±1.7	3.0 ±2.2	.14
ACP classification: class I	44.8	47.2	42.5	.52
class II	28.9	22.2	35.0	
class III	17.1	22.2	12.5	
class IV	9.2	8.3	10.0	
Denture quality: acceptable	68.4	58.3	77.5	.07
unacceptable	31.6	41.7	22.5	
Peanut particle size (mm): mean ±SD	2.2 ±1.1	2.4 ±1.2	2.0 ±1.0	.11
Oral impact at T0: absence	51.3	22.2	77.5	<.001
presence	48.7	77.8	22.5	

†Analyzed using Chi-squared test or independent t-test.

The participants who continued using DA at T2 more frequently presented an oral impact at T0 compared with those who discontinued using DA, regardless of CD quality (Table 2). At T0, a major problem was having an eating impact, while a cleaning impact was absent. Eating, speaking, and emotional impacts at T1 significantly decreased in the participants who continued using DA at T2. In contrast, emotional and cleaning impacts at T1 significantly emerged in the participants who discontinued using DA. The prevalence of an oral impact at T2 in the acceptable CD wearers who discontinued DA use was significantly decreased from baseline.

Table 2. Percentage of the participants who had oral impact at each time point.

Oral impact	Oral impact at T0	Presence (N=37)				Absence (N=39)			
	CD quality	Acceptable (n=23)		Unacceptable (n=14)		Acceptable (n=29)		Unacceptable (n=10)	
	DA use at T2	Continue (n=15)	Discontinue (n=8)	Continue (n=13)	Discontinue (n=1)†	Continue (n=6)	Discontinue (n=23)	Continue (n=2)†	Discontinue (n=8)
**Overall**	**T0**	100.0^{a}	100.0^{a}	100.0^{a}	100.0	0.0	0.0^{b}	0.0	0.0
	**T1**	53.3^{b}	62.5^{a}	15.4^{b}	100.0	0.0	52.2^{a}	50.0	37.5
	**T2**	20.0^{b}	12.5^{b}	30.8^{b}	100.0	0.0	0.0^{b}	0.0	12.5
**Physiological**									
**Eat**	**T0**	93.3^{a}	87.5^{a}	100.0^{a}	100.0	0.0	0.0	0.0	0.0
	**T1**	26.7^{b}	37.5^{b}	15.4^{b}	100.0	0.0	13.0	0.0	0.0
	**T2**	6.7^{b}	12.5^{b}	15.4^{b}	100.0	0.0	0.0	0.0	0.0
**Speak**	**T0**	33.3^{a}	0.0	53.9^{a}	100.0	0.0	0.0	0.0	0.0
	**T1**	6.7^{b}	12.5	0.0^{b}	100.0	0.0	0.0	0.0	12.5
	**T2**	6.7^{b}	0.0	0.0^{b}	100.0	0.0	0.0	0.0	12.5
**Clean**	**T0**	0.0^{b}	0.0^{b}	0.0	0.0	0.0	0.0^{b}	0.0	0.0
	**T1**	26.7^{a}	50.0^{a}	0.0	100.0	0.0	43.5^{a}	50.0	37.5
	**T2**	20.0^{a}	0.0^{b}	7.7	0.0	0.0	0.0^{b}	0.0	0.0
**Psychological**									
**Emotion**	**T0**	6.7	0.0^{b}	23.1	100.0	0.0	0.0^{b}	0.0	0.0
	**T1**	13.3	25.0^{a}	0.0	100.0	0.0	17.4^{a}	0.0	0.0
	**T2**	13.3	0.0^{b}	7.7	100.0	0.0	0.0^{b}	0.0	0.0
**Smile**	**T0**	13.3^{a}	12.5	15.4	0.0	0.0	0.0	0.0	0.0
	**T1**	0.0^{b}	0.0	0.0	0.0	0.0	0.0	0.0	0.0
	**T2**	0.0^{b}	0.0	0.0	0.0	0.0	0.0	0.0	0.0

**Sleep**	**T0**	6.7	0.0	0.0	0.0	0.0	0.0	0.0	0.0
	**T1**	6.7	12.5	0.0	0.0	0.0	4.4	0.0	0.0
	**T2**	6.7	0.0	0.0	0.0	0.0	0.0	0.0	0.0
**Social**									
**Contact**	**T0**	0.0	12.5	0.0	0.0	0.0	0.0	0.0	0.0
	**T1**	0.0	0.0	0.0	0.0	0.0	0.0	0.0	0.0
	**T2**	0.0	0.0	0.0	0.0	0.0	0.0	0.0	0.0
**Work**	**T0**	0.0	12.5	0.0	0.0	0.0	0.0	0.0	0.0
	**T1**	0.0	0.0	0.0	0.0	0.0	0.0	0.0	0.0
	**T2**	0.0	0.0	0.0	0.0	0.0	0.0	0.0	0.0

Different alphabetical letters indicated significant difference between DA use within the same time point.

[†]No statistical analysis between time points due to a very low number of sample (n=1, 2).

Peanut particle size was evaluated at each time point, a smaller peanut particle size indicated higher masticatory performance (Table 3). Among the participants with an oral impact, the peanut particle size at T0 tended to be larger in CD wearers who continued using DA, compared with that of the participants who discontinued using DA. A significant reduction in peanut particle size from T0 to T2 was present only in the acceptable CD wearers who discontinued using DA. A positive correlation between peanut particle size and an eating impact score was found only at T0 (rho = 0.30, p=0.008), but not at T1 or T2 (rho = 0.08, p=0.478).

Table 3. Peanut particle size (mm; mean ±SD) at each time point.

**Oral impact at T0**		**Presence (N=37)**				**Absence (N=39)**			
**CD quality**		**Acceptable (n=23)**		**Unacceptable (n=14)**		**Acceptable (n=29)**		**Unacceptable (n = 10)**	
**DA use at T2**		**Continue (n=15)**	**Discontinue (n=8)**	**Continue (n=13)**	**Discontinue[†] (n=1)**	**Continue (n=6)**	**Discontinue (n=23)**	**Continue[†] (n=2)**	**Discontinue (n=8)**
**Time:**	**T0**	2.37 ±1.26	2.08 ±0.92[a]	2.93 ±1.28	1.53 (N/A)	1.78 ±0.78	1.90 ±1.12	1.70 ±0.95	2.37 ±0.97
	**T1**	2.04 ±0.82	1.87 ±0.67[a]	2.46 ±0.83	1.14 (N/A)	1.42 ±0.48	1.71 ±0.93	2.58 ±0.48	1.85 ±1.44
	**T2**	2.35 ±0.91	1.52 ±0.61[b]	2.46 ±0.92	1.32 (N/A)	1.43 ±0.31	1.43 ±0.31	2.11 ±0.92	2.23 ±1.83

N/A, not applicable. Different small letters indicate significant difference between time points within the same column (p<0.05).
[†]No statistical analysis between decision of DA use and time points due to a very low number of samples in the subgroup (n=1, 2).

Discussion

The present study provided the 1-month trial period of DA use for CD wearers and allowed the patients to decide whether to continue using DA. The results showed that the CD wearers who continued using DA at T2 had a greater oral impact reduction after a 1-month trial of DA use, compared with the patients who discontinued using DA. In contrast, masticatory performance change after 1-month of DA use between the CD wearers who continued and discontinued using DA was not significantly different. To mimic a pragmatic trial of real-world clinical use, DA was provided for both the acceptable and unacceptable CD wearers but its use was limited to 1–2 months within our study duration. After the study ended, DA use in unacceptable CD wearers was prohibited and all of them underwent a new CD fabrication.

The present study used the OIDP for an OHRQoL evaluation. Previous studies evaluated the effect of DA use on the OHRQoL of CD wearers by using the Oral Health Impacts Profile for Edentulous Patients (OHIP-EDENT) [12], short-formed Oral Health Impacts Profile (OHIP-14) [24], or Geriatric Oral Health Assessment Index (GOHAI) [25]. However, the OHIP and GOHAI evaluation do not include cleaning oral mucosa and denture, which is a major problem caused by DA use. Thus, an additional questionnaire had to be included to detect cleaning problem [7]. Based on the fact that the OIDP covers a cleaning aspect, our study recommended it as a subjective indicator for evaluating an impact of DA use on the OHRQoL of CD wearers.

Our findings revealed that presence of an oral impact and its changes after a 1-month trial of DA use influenced the decision to continue using DA in CD wearers. In CD wearers who decided to continue DA use, eating and speaking impacts were generally decreased, while a

cleaning impact significantly emerged after the 1-month of DA use. Despite decreasing eating and speaking impacts, the decision to discontinue using DA was commonly due to an emerging cleaning impact. At baseline, an emotional disturbance was caused by an eating impact; however, at T1, it was caused by a cleaning impact. As found in previous studies in CD wearers [2, 7, 14], common reasons for stopped using DA are its difficulty to remove from the denture and oral mucosa, and an unpleasant taste and texture. Therefore, a patient's decision to use DA may depend on an emerging oral impact caused by DA that is more troublesome compared with the problems solved by DA use. Due to this cleaning problem, DA use may not be suitable for CD wearers who have poor manual dexterity or denture hygiene practice.

The masticatory performance of CD wearers and its change after 1-month of DA use did not significantly influence the decision to continue using DA. Previous studies reported that DA use in newly-fabricated CD wearers significantly improved their masticatory performance [6, 26-28], both in normal and resorbed ridges [27]. However, our study found no masticatory performance improvement after 1-month of DA use, either in well-fitting or ill-fitting denture, or in any patients' condition based on ACP classification. A significant association between the presence of an eating impact and lower masticatory performance was observed at baseline, but not at T1 or T2. A patient-reported oral impact might be more sensitive to changes caused by DA use compared with an objective masticatory performance, which can be influenced by several factors, such as patient's age, and the occlusal contact area of the artificial teeth [4]. Also, it might be possible that the patients with ill-fitting denture had already accommodated and learnt to manipulate the denture.

Professional evaluation of CD retention and stability did not affect the patients' decision to continue using DA. However, approximately 42% of CD wearers who continued using DA possessed unacceptable CD retention and stability, and DA use significantly reduces the oral impacts of these CD wearers. It is known that DA should not be used in ill-fitting denture because it would compromise the adaptation between the underlying tissue and a denture, leading to denture supporting tissue traumatization without the patients' awareness. Because DA is an over-the counter product, its application under unsuitable oral or denture condition could be harmful to the CD wearers without patients' notice. Therefore, DA use would rather be prescribed by a dental professional after a denture retention and stability evaluation. In addition, dentists should provide a maintenance recall for periodic check-up of denture quality to prevent inappropriate DA use in CD wearers.

One interesting finding was present in the acceptable CD wearers who had an oral impact at baseline but discontinued using DA at T2. In this subgroup, their OHRQoL and masticatory performance continuously improved from baseline to T1 and T2, despite discontinued DA use at T2. In contrast with a previous pilot study in well-fitting CD wearers, the patients who continued using DA for 6 months demonstrated greater OHRQoL improvement and slightly higher masticatory parameters, compared with those who discontinued using DA at 3 months [10]. Our findings imply that a 1-month trial of DA use may assist the acceptable CD wearers to control and adapt to their denture during function. In acceptable CD wearers with a persisting oral impact, other treatment options might be considered, such as an implant-retained mandibular overdenture [29].

The present study found no significant associations between decision to continue using DA and patients' age, sex, previous CD experience, denture age, or the patient's ACP classification. Previous cross-sectional surveys in removable denture wearers demonstrated the association between DA use and patient-related factors rather than denture characteristics. They concluded that DA use was more common in smokers, people who regularly visit a dental clinic [13], had been wearing the denture for a shorter time, and had a lower frequency of denture use [17]. However, many denture wearers have never tried using DA due to a lack of DA knowledge and being unaware that DAs are available. Unless CD wearers have tried using DA, patient-related conditions and CD experience might not be the appropriate indicator to predict whether CD wearers would prefer DA use.

From our findings, the three considerations for identifying the CD wearers who would benefit from DA use were a professional assessment of CD retention and stability, a patient-reported baseline oral impact, and oral impact changes after a 1-month trial of DA use. With the presence of an oral impact, the unacceptable CD wearers would not be eligible for DA use, whereas the acceptable-quality CD wearers might undergo a 1-month trial of DA use. The oral impact should be then re-evaluated. The patients would decide whether to continue using DA based on the emerging and decreasing oral impacts. Despite discontinuing DA use, the temporary use of DA for 1 month may assist some well-fitting CD wearers in adapting to their denture.

The present study has some limitations. First, only a cream-type DA was provided to the patients, because it is the most available commercial form in Thailand, whereas a powder-type is limited to professional use in dental clinics. Second, it remains unidentified whether the patients

would continue using DA based on the additional cost of DA. Further studies with a longer follow-up period are recommended for better understanding the effect of DA use in CD wearers and their adaptability to DA use. A cost-benefit analysis should be conducted to evaluate all potential costs and revenues that generate from long-term DA use.

Conclusions

Within the limitations of this clinical study, it can be concluded that presence of oral impact at baseline and its change influenced the CD wearers' decision whether to continue using DA. Meanwhile, masticatory performance at baseline and its change showed no influence on patients' decision to continue using DA.

Acknowledgments

The authors would like to gratefully acknowledge Dr.Kevin Tompskin for language revision of the manuscript.

Funding

The present research was funded by the Faculty Research Grant, Faculty of Dentistry, Chulalongkorn University [grant number DRF 63002]. The funders had no role in study design, data collection and analysis, decision to publish, or preparation of the manuscript.

Conflict of interest

The authors declare that there is no potential conflict of interest in this study.

Data availability statement

All relevant data are within the paper and the supporting information file.

Supporting information file

S1. CONSORT 2010 checklist information.

S2. Raw data of the present study.

S3. Study protocol in Thai.

S4. Study protocol in English.

References

[1] Carlsson G E,Omar R. The future of complete dentures in oral rehabilitation. A critical review. J Oral Rehabil 2010;37(2):143-156.

[2] Coates A J. Usage of denture adhesives. J Dent 2000;28(2):137-140.

[3] Limpuangthip N, Somkotra T,Arksornnukit M. Modified retention and stability criteria for complete denture wearers: A risk assessment tool for impaired masticatory ability and oral health-related quality of life. J Prosthet Dent 2018;120(1):43-49.

[4] Limpuangthip N, Somkotra T,Arksornnukit M. Subjective and objective measures for evaluating masticatory ability and associating factors of complete denture wearers: A clinical study. J Prosthet Dent 2021;125(2):

[5] Ozcan M, Kulak Y, Arikan A,Silahtar E. The attitude of complete denture wearers towards denture adhesives in Istanbul. J Oral Rehabil 2004;31(2):131-134.

[6] Shu X, Fan Y, Lo E C M,Leung K C M. A systematic review and meta-analysis to evaluate the efficacy of denture adhesives. J Dent 2021;108(103638.

[7] Kulak Y, Ozcan M,Arikan A. Subjective assessment by patients of the efficiency of two denture adhesive pastes. J Prosthodont 2005;14(4):248-252.

[8] Munoz C A, Gendreau L, Shanga G, Magnuszewski T, Fernandez P,Durocher J. A clinical study to evaluate denture adhesive use in well-fitting dentures. J Prosthodont 2012;21(2):123-129.

[9] Atassi M, Milleman K R, Burnett G R, Sanyal S,Milleman J L. A randomized clinical study to evaluate the effect of denture adhesive application technique on food particle accumulation under dentures. Clin Exp Dent Res 2019;5(4):316-325.

[10] Nicolas E, Veyrune J L,Lassauzay C. A six-month assessment of oral health-related quality of life of complete denture wearers using denture adhesive: a pilot study. J Prosthodont 2010;19(6):443-448.

[11] Fujimori T, Hirano S,Hayakawa I. Effects of a denture adhesive on masticatory functions for complete denture wearers--consideration for the condition of denture-bearing tissues. J Med Dent Sci 2002;49(4):151-156.

[12] Ohwada G, Minakuchi S, Sato Y, Kondo H, Nomura T, Tsuboi A, et al. Subjective Evaluation of Denture Adhesives: A Multicenter Randomized Controlled Trial. JDR Clin Trans Res 2019;2380084419837607.

[13] Bo T M, Hama Y, Akiba N,Minakuchi S. Utilization of denture adhesives and the factors associated with its use: a cross-sectional survey. BMC Oral Health 2020;20(1):194.

[14] Shah R J, Lagdive S B, Talkal A K, Agrawal H,Darji B. Knowledge and attitude toward denture adhesives: A survey on dentists and complete denture wearers. Int J Prosthodont Restor Dent 2015;5(3):74-80.

[15] Al Taweel S M,Al Shehri H A. Knowledge and attitudes of dental interns toward denture adhesives in King Saud University, Riyadh, Saudi Arabia. Eur J Dent 2016;10(4):536-540.

[16] Polyzois G, Lagouvardos P, Omar R,Brunton P. Attitudes of dentists toward denture adhesives: A questionnaire survey in Greece. J Prosthet Dent 2017;118(5):643-649.

[17] Kossioni A E. Prevalence and Factors Associated with the Use of Denture Adhesives by Older Complete Denture Wearers. Eur J Prosthodont Restor Dent 2018;26(4):197-201.

[18] McGarry T J, Nimmo A, Skiba J F, Ahlstrom R H, Smith C R,Koumjian J H. Classification system for complete edentulism. The American College of Prosthodontics. J Prosthodont 1999;8(1):27-39.

[19] Limpuangthip N, Somkotra T,Arksornnukit M. Impacts of denture retention and stability on oral health-related quality of life, general health, and happiness in elderly Thais. Current Gerontology and Geriatrics Research 2019;2019(8.

[20] Srisilapanan P,Sheiham A. The prevalence of dental impacts on daily performances in older people in Northern Thailand. Gerodontology 2001;18(2):102-108.

[21] Kapur K K,Soman S D. Masticatory performance and efficiency in denture wearers. 1964. J Prosthet Dent 2004;92(2):107-111.

[22] Grasso J E. Denture adhesives. Dent Clin North Am 2004;48(3):721-733, vii.

[23] Felton D, Cooper L, Duqum I, Minsley G, Guckes A, Haug S, et al. Evidence-based guidelines for the care and maintenance of complete dentures: a publication of the American College of Prosthodontists. J Prosthodont 2011;20 Suppl 1(S1-S12.

[24] Polyzois G, Lagouvardos P, Partalis C, Zoidis P,Polyzois H. Short-Term Assessment of the OHIP-14 Scale on Denture Wearers Using Adhesives. J Prosthodont 2015;24(5):373-380.

[25] Abdelnabi M H, Swelem A A,Al-Dharrab A A. Influence of denture adhesives on occlusion and disocclusion times. J Prosthet Dent 2016;115(3):306-312.

[26] de Oliveira Junior N M, Rodriguez L S, Mendoza Marin D O, Paleari A G, Pero A C,Compagnoni M A. Masticatory performance of complete denture wearers after using two adhesives: a crossover randomized clinical trial. J Prosthet Dent 2014;112(5):1182-1187.

[27] Goncalves T M, Viu F C, Goncalves L M,Garcia R C. Denture adhesives improve mastication in denture wearers. Int J Prosthodont 2014;27(2):140-146.

[28] Torres-Sanchez C, Montoya-Salazar V, Torres-Lagares D, Gutierrez-Perez J L,Jimenez-Castellanos E. Comparison of masticatory efficacy among complete denture wearers with two adhesives and dentate individuals: A randomized, crossover, double-blind clinical trial. J Prosthet Dent 2017;117(5):614-620.

[29] Feine J S, Carlsson G E, Awad M A, Chehade A, Duncan W J, Gizani S, et al. The McGill consensus statement on overdentures. Mandibular two-implant overdentures as first choice standard of care for edentulous patients. Gerodontology 2002;19(1):3-4.

Figure 1 consort flow

Click here to access/download;Figure;Figure DA plosone_convert.jpg

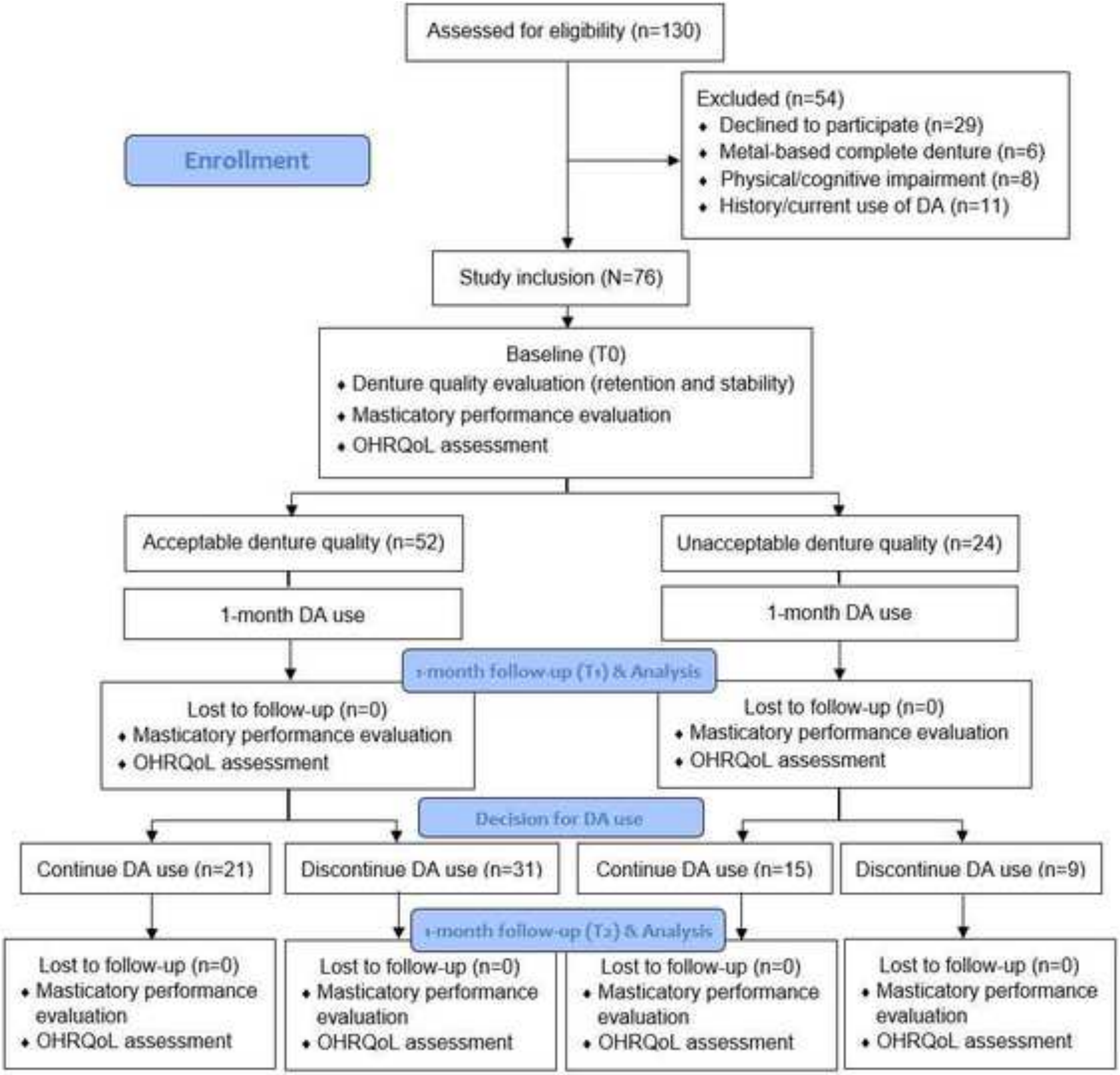

S2 Raw data

Click here to access/download
Supporting Information
S2_RawData_DA.xlsx

S1 Consort checklist

Click here to access/download
Supporting Information
S1_CONSORT 2010 Checklist_DA.docx

S3 Ethical protocol in Thai

Click here to access/download
Supporting Information
S3_ETHIC_DA study protocol_Thai.pdf

S4 Ethical protocol in English translation

Click here to access/download

Supporting Information

S4_ETHIC_DA study protocol_Eng.pdf
