# Supplementary material for: Decision to use denture adhesive in complete denture wearers after one-month run-in period: A quasi-experimental study
Source: PLoS One. 2022 Dec 1;17(12):e0276760. doi: 10.1371/journal.pone.0276760 (PMC9714889; doi:10.1371/journal.pone.0276760)
Supplement: Attachment — Submitted filename: Revise2_Response to reviewers.docx [file pone.0276760.s007.docx]

**Response to reviewer**

The authors are pleased to submit our revised manuscript ID. PONE-D-22-08584R1, entitled ‘Decision to Use Denture Adhesive in Complete Denture Wearers After One-Month Run-In Period: A Quasi-Experimental Study’. The authors have changed the sequence of reference lists corresponding to the previous changes at first revision. The requested revisions have been made in the manuscript in track changes, and our point-by-point responses are below.

**Reviewer #1:**

Comment: The authors made significant improvements in their manuscript. I am satisfied with the present version.

Response: The authors would like to thank you for reviewers’ comments which make the substantial improvement of the manuscript.

**Reviewer #2:**

Comment: Minor revision (Note that line numbers refer to those in the tracked changes version.)
Lines 174-6: In the "Statistical analysis" section, describe only the statistical methods. Do not include the results. Remove the fact that an interaction effect was found. Simply indicate that an interaction effect was tested.

Response: In the statistical analysis section, the sentence has been revised to ‘The effect of denture quality and the decision to use DA on peanut particle size across time points were assessed using two-way repeated measures ANOVA, and an interaction effect of denture quality and time was tested.’

Sincerely yours,

Wacharasak Tumrasvin

Corresponding author
